# Voltage‐gated sodium channels in genetic epilepsy: up and down of excitability

**DOI:** 10.1111/jnc.15947

**Published:** 2023-08-31

**Authors:** Evgeniia Rusina, Martina Simonti, Fabrice Duprat, Sandrine Cestèle, Massimo Mantegazza

**Affiliations:** ^1^ University Cote d'Azur Valbonne‐Sophia Antipolis France; ^2^ CNRS UMR 7275 Institute of Molecular and Cellular Pharmacology (IPMC) Valbonne‐Sophia Antipolis France; ^3^ Inserm Valbonne‐Sophia Antipolis France

**Keywords:** developmental and epileptic encephalopathies, ion channels, migraine, movement disorders, neurodevelopmental diseases, seizures

## Abstract

The past two decades have witnessed a wide range of studies investigating genetic variants of voltage‐gated sodium (Na_V_) channels, which are involved in a broad spectrum of diseases, including several types of epilepsy. We have reviewed here phenotypes and pathological mechanisms of genetic epilepsies caused by variants in Na_V_ α and β subunits, as well as of some relevant interacting proteins (*FGF12/FHF1*, PRRT2, and Ankyrin‐G). Notably, variants of all these genes can induce either gain‐ or loss‐of‐function of Na_V_ leading to either neuronal hyperexcitability or hypoexcitability. We present the results of functional studies obtained with different experimental models, highlighting that they should be interpreted considering the features of the experimental system used. These systems are models, but they have allowed us to better understand pathophysiological issues, ameliorate diagnostics, orientate genetic counseling, and select/develop therapies within a precision medicine framework. These studies have also allowed us to gain insights into the physiological roles of different Na_V_ channels and of the cells that express them. Overall, our review shows the progress that has been made, but also the need for further studies on aspects that have not yet been clarified. Finally, we conclude by highlighting some significant themes of general interest that can be gleaned from the results of the work of the last two decades.

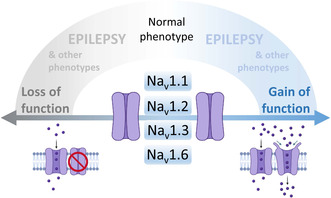

## Graphical abstract

Genetic variants of Na_V_1.1 (*SCN1A*), Na_V_1.2 (*SCN2A*), Na_V_1.3 (*SCN3A*), and Na_V_1.6 (*SCN8A*) sodium channels are involved in a broad spectrum of diseases, including several types of epilepsy. In this review, we describe the functional effect of variants of Na_V_ and their interacting proteins, and their correlation to different phenotypes. They can induce either gain‐ or loss‐of‐function of Na_V_ leading to either neuronal hyperexcitability or hypoexcitability, which are often neuronal‐subtype specific. However, detailed pathological mechanisms are not always clear and need to be further investigated. We highlighted the importance of functional studies and investigations in animal models to improve diagnosis and to develop therapeutic strategies.

**Abbreviations**
AIS: axonal initial segment
APs: action potentials
ASD: autism spectrum disorders
ASOs: antisense oligonucleotides
BD: bipolar disorder
DEEs: Developmental and Epileptic Encephalopathies
DS: Dravet syndrome
EIDEE: early infantile DEE without movement disorder
EIDEE/MD: early infantile developmental and epileptic encephalopathy with hyperkinetic movement disorder
FGFs: fibroblast growth factors
FHM: familial hemiplegic migraine
FS: febrile seizures
iPSC: induced pluripotent stem cell
LOF: loss of function
NDEEMA: epileptic encephalopathy with hyperkinetic movement disorder and arthrogryposis
PKD: Paroxysmal Kinesigenic Dyskinesia
PRRT2: Proline‐rich transmembrane protein‐2
SUDEP: sudden unexpected death in epilepsy
TLE‐HS: temporal lobe epilepsy with hippocampal sclerosis

## 1 INTRODUCTION

Voltage‐gated Na^+^ channels (Na_V_) are essential for neuronal excitability because they generate the depolarizing Na^+^ currents that initiate and propagate action potentials (APs) (Hodgkin & Huxley, 1952). Na^+^ currents have fast activation and open upon depolarization of the plasma membrane. They are transient because they inactivate within few milliseconds after activation, although a small fraction can persist for longer periods during depolarizations (persistent current); both transient and persistent current undergo slow inactivation with kinetics of hundreds of ms (Mantegazza et al., 2021). The core molecular Na_V_ complex is composed of a principal pore‐forming α subunit (nine isoforms: Na_V_1.1‐Na_V_1.9 for the proteins, *SCN1A*‐*SCN11A* for the genes) and of auxiliary β subunits that modulate the properties of the α subunit (four β subunit isoforms: β1–β4 for the proteins, *SCN1B*‐*SCN4B* for the genes) (Bouza & Isom, 2018; Mantegazza et al., 2021; https://www.guidetopharmacology.org/GRAC/FamilyDisplayForward?familyId=82). The primary sequence of the α subunits contains four homologous domains (DI‐DIV), each comprising six transmembrane segments (S1–S6). In each domain, S1–S4 form the voltage‐sensing module (S4 contains the voltage‐sensing charges), responsible for the voltage‐dependent properties, and S5–S6 with their connecting extracellular loop form the pore module, responsible for ion conduction (Catterall et al., 2020). The β subunits contain a single transmembrane segment (Bouza & Isom, 2018). Recent studies have revealed the detailed structure of the core molecular Na_V_ complex (Catterall et al., 2020; Pan et al., 2019). Na_V_ are clustered at high density at the axonal initial segment (AIS), which is, for this reason, the primary site for generation of APs in neurons (Rasband, 2010). In myelinated axons, Na^+^ channels are also clustered at high density in Ranvier's nodes, which allows saltatory axonal conduction (Boiko et al., 2001; Kaplan et al., 2001). The core Na_V_ complex can interact with numerous other proteins that are important for trafficking, localization, and modulation of functional properties (Curran & Mohler, 2015; Dib‐Hajj & Waxman, 2010; Terragni et al., 2018). Moreover, it has been proposed that Na_V_ α subunits can dimerize (Clatot et al., 2017), but more studies are necessary to validate this mechanism and understand its functional role. Notably, Na_V_s are targets of drugs used in different clinical settings, the sodium channel blockers, which include several anti‐seizure medications (Catterall et al., 2020; Mantegazza, Curia, et al., 2010).

Na_V_s are involved in numerous diseases. In this article, we focus on their involvement in genetic epilepsy. In fact, pathogenic variants of *SCN1A*/Na_V_1.1, *SCN2A*/Na_V_1.1, *SCN3A*/Na_V_1.1, *SCN8A*/Na_V_1.6, and *SCN1B*/β1, which are expressed in the central nervous system, are important causes of different types of epilepsies, including mild and severe forms (Brunklaus & Lal, 2020; Mantegazza et al., 2021; Meisler et al., 2021). Severe forms have in general features of Developmental and Epileptic Encephalopathies (DEEs) (Guerrini et al., 2023), which are characterized by early‐onset severe epileptic seizures and EEG abnormalities on a background of developmental impairment that tends to worsen as a consequence of epilepsy (Scheffer et al., 2017). The identification of a genetic variant in a patient is often just the beginning of a complex task. In fact, predictive tools have limited efficacy in predicting the effects of variants on protein's functions. Experimental functional analysis and investigations in animal models are essential to disclose detailed functional effects and pathological mechanisms (Guerrini et al., 2023; Mantegazza, Rusconi, et al., 2010), which should be integrated into precision medicine pipelines to improve diagnosis and develop effective precision therapies (Mantegazza & Cestele, 2022).

In vitro experimental systems are either cells that usually do not express the protein of interest (often human cell lines, e.g., human embryonic kidney cells), which simplify the functional analysis, or neurons in primary cultures, which provide neuronal cellular background and variety but more complex experimental conditions. In vivo/ex vivo systems are organisms (often gene‐targeted mice or, for high‐throughput experiments, zebrafish) or preparations obtained from them (e.g., brain slices), which should better model the complexity of brain circuits and pathophysiological conditions (including secondary responses, e.g., homeostatic). Human neurons (which conserve the genetic background of a given patient) can be obtained by differentiating human neurons from induced pluripotent stem cells generated from biopsies of patients. Overall, it is important that the results of functional studies are interpreted considering the features of the experimental system used (Guerrini et al., 2023; Mantegazza, Rusconi, et al., 2010).

## 2 PRINCIPAL α SUBUNITS

### 2.1 *SCN1A* /Na_V_1.1

**FIGURE 1:**
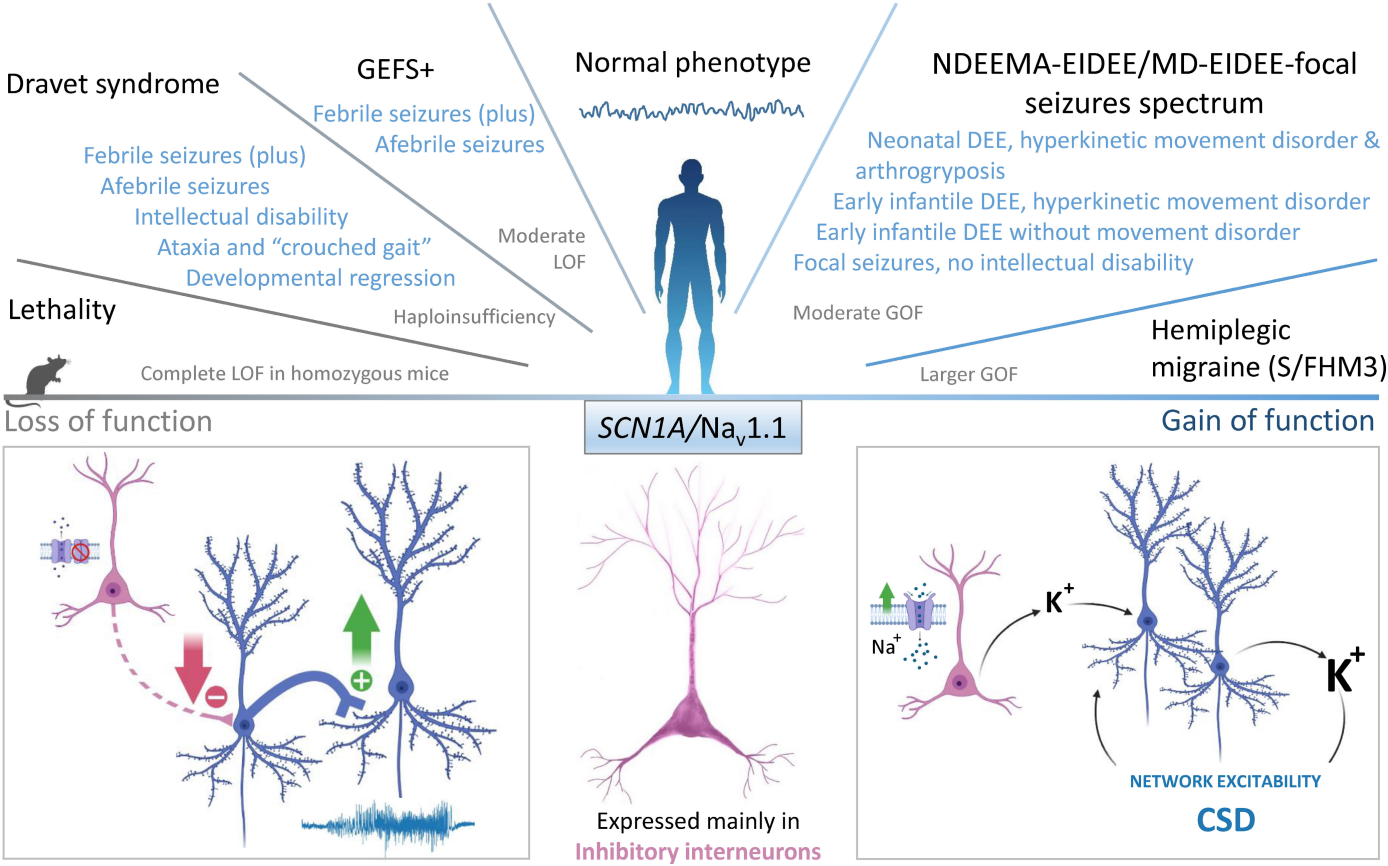
*SCN1A*/Na_v_1.1 variants: clinical phenotypes and functional effects. *Top panel*: phenotypic spectrum of *SCN1A* variants with correlation to functional effects. *Bottom panel*: (Middle) Na_v_1.1 is mainly expressed in inhibitory interneurons. (Left) Loss of Na_v_1.1 function in inhibitory interneurons reduces GABAergic inhibition, leading to network hyperexcitability. (Right) Gain of Na_v_1.1 function in inhibitory interneurons can induce their hyperactivation, and increases spike‐generated extracellular K^+^ triggering positive feedback: spiking of depolarized excitatory neurons contributes to extracellular K^+^ increase, leading to network hyperexcitability and cortical spreading depolarization. This mechanism has been proposed for S/FHM3 variants; the detailed pathological mechanism of NDEEMA‐EIDEE/MD‐EIDEE‐focal seizure variants is not clear yet.

*SCN1A*, encoding the Na_V_1.1 α subunit, is one of the most clinically relevant epilepsy genes, with thousands of genetic variants reported thus far in different phenotypes (McTague et al., 2016; Mei et al., 2019; Scheffer & Nabbout, 2019) (Figure 1).

#### 2.1.1 Phenotypic spectrum

Heterozygous missense *SCN1A*/Na_V_1.1 genetic variants can cause GEFS+, a familial epilepsy syndrome characterized by a distinctive pattern of pleiotropy in clinical phenomenology (Escayg et al., 2000; Scheffer & Berkovic, 1997; Zhang et al., 2017): the most frequent phenotype is febrile seizures plus (FS+: febrile/hyperthermic seizures that extend beyond 6 years of age) and afebrile generalized tonic–clonic seizures. Less common phenotypes include FS/FS+ with absence, myoclonic, atonic, or focal seizures, and at the severe end of the spectrum myoclonic–atonic seizures and the DEE Dravet syndrome (DS).

Following the identification of GEFS+ variants, de novo heterozygous *SCN1A*/Na_V_1.1 variants have been identified in DS patients (Claes et al., 2001; Dravet, 2011), which is one of the most studied DEE (Guerrini et al., 2023). They can be either missense or give rise to a truncated in general non‐functional protein. The clinical spectrum of DS does not have firmly established boundaries, but the core phenotype is characterized by clonic seizures precipitated by increased body temperature (often febrile status epilepticus) with onset between 6 months and 1 year of age, and subsequent appearance of multiple other seizure types, both hyperthermia‐induced and hyperthermia‐independent. Development is normal in the first year of life but plateaus rapidly, with most patients showing cognitive impairment when the full syndrome is manifested, as well behavioral dysfunctions, ataxia, gait deterioration, and sudden unexpected death in epilepsy (SUDEP). Pathogenic variants of other genes can cause DS‐like phenotypes (including *SCN1B*, *HCN1*, *KCN2A*, *GABRA1*, *GABRG2*, and *STXBP1*), but they induce their own, different, and specific clinical picture (Steel et al., 2017). Thus, the association between *SCN1A*/Na_V_1.1 pathogenic variants and DS is highly specific (Guerrini et al., 2023).

*SCN1A* variants have also been implicated in simple febrile seizures (FS), which are convulsive seizures triggered by fever occurring between 6 months and 6 years of age. Although they are self‐limited, around 3%–8% of children with FS may later develop epilepsy, in particular, temporal lobe epilepsy with hippocampal sclerosis (TLE‐HS) (Patel et al., 2015). Genetic loci have been identified in population studies of FS patients and one of them, FEB3, contains *SCN1A* (Baulac et al., 2004). Interestingly, an *SCN1A* loss of function (LOF) missense variant has been identified in a large family with simple FS and development of TLE‐HS in three of the 14 patients (Colosimo et al., 2007; Mantegazza et al., 2005). It has been proposed to include this family in the GEFS+ spectrum (Mullen & Scheffer, 2009), but FS are not self‐limited in GEFS+ or DS patients, and they do not develop TLE‐HS (Catarino et al., 2011; Guerrini et al., 2011). Consistently, animal models of DS or GEFS+ carrying LOF *Scn1a*/Na_V_1.1 epilepsy variants do not show noticeable neuronal death (Ogiwara et al., 2007; Salgueiro‐Pereira et al., 2019; Yu et al., 2006). Interestingly, genome‐wide association studies have linked common *SCN1A* single nucleotide polymorphisms to development of TLE‐HS upon a history of FS (Kasperaviciute et al., 2013).

Missense *SCN1A*/Na_V_1.1 variants can also cause sporadic/familial hemiplegic migraine type3 (S/FHM3), a rare form of migraine with aura with onset in adolescence, characterized by hemiparesis as part of the aura phase (Dichgans et al., 2005; Ferrari et al., 2015; Mantegazza & Cestele, 2018).

Moreover, missense *SCN1A*/Na_V_1.1 variants have been recently identified in a spectrum of epileptic phenotypes that do not fit the clinical features outlined above (Brunklaus et al., 2022; Matricardi et al., 2023; Sadleir et al., 2017). The most severe phenotypes in the spectrum are caused by de novo variants: neonatal developmental and epileptic encephalopathy with hyperkinetic movement disorder and arthrogryposis (NDEEMA), which is followed in severity by an early infantile developmental and epileptic encephalopathy with hyperkinetic movement disorder (EIDEE/MD, with onset in the first weeks or the first few months of age) and by an early infantile DEE without movement disorder (EIDEE, with in general later onset in the first months of life). Sporadic or familial focal epilepsies with mild or no intellectual disability are the least severe phenotypes in the spectrum.

#### 2.1.2 Functional features of LOF variants

Since the identification of truncating *SCN1A*/Na_V_1.1 variants in DS patients, their functional effect has thought to be haploinsufficiency: 50% reduction of functional Na_V_1.1 protein in heterozygotes, with complete LOF for the mutant allele (Claes et al., 2001). This mechanism has been subsequently verified in functional studies in in vitro expression systems (transfected cell lines) (Bechi et al., 2012). The effect of DS and GEFS+ missense variants studied in vitro has been more controversial, but with most of the results showing a loss of function, whose severity loosely correlates with the severity of the phenotype, although some variants do not show this correlation (Bryson & Petrou, 2023; Mantegazza et al., 2021; Mantegazza & Broccoli, 2019; Meisler et al., 2021). Thus, it has been proposed that the severity spectrum of Na_V_1.1‐related epilepsies could be a continuum and depend on the amount of LoF of the mutant: a mild impairment of Na_V_1.1 function would cause mild phenotypes, whereas a more complete LOF would cause severe phenotypes. Interestingly, some Na_V_1.1 missense mutations cause LOF because of folding/trafficking defects that lead to channel degradation in intracellular compartments (Terragni et al., 2018); these mutants can often be rescued by interacting proteins that probably stabilize the correct folding conformation (Bechi et al., 2015; Rusconi et al., 2007, 2009; Thompson et al., 2012). In vivo, similar interactions of Na_v_1.1 with other proteins may regulate the LOF and modulate with this mechanism the phenotype, explaining at least in part the not perfect correlation, with can be altered more broadly by the genetic background.

#### 2.1.3 Animal models of LOF variants

It initially seemed puzzling that mutations involved in epileptic phenotypes could lead to LOF and reduced Na^+^ current, consistent with reduced neuronal excitability. Gene‐targeted mouse models have shed light on this issue. The first model generated was a global knockout that modeled a truncating DS mutation (Yu et al., 2006). Heterozygous *Scn1a* ^+/−^ mice showed seizures (including hyperthermia‐induced ones) and sporadic deaths beginning at postnatal day (P)21. It was observed that the Na^+^ current density was reduced without modifications of gating properties in GABAergic interneurons, causing their hypoexcitability, but not in glutamatergic excitatory neurons. This suggested that the decreased excitability of GABAergic interneurons, induced by DS Na_V_1.1 epileptogenic variants, could reduce GABAergic inhibition and cause network hyperexcitability: it was one the first clear identifications of the pathologic mechanism of a DEE and the identification of Na_V_1.1 as the predominant Na^+^ channel of GABAergic interneurons. This study has shown also a clear influence of the genetic background on the phenotype, which has been later investigated in cellular details (Rubinstein et al., 2015). Another early study with a knock‐in model expressing a truncating nonsense DS mutation reported a similar phenotype, showing that Na_V_1.1 localizes to the AIS of GABAergic interneurons, in particular fast‐spiking parvalbumin (PV)‐positive ones (Ogiwara et al., 2007). Subsequent studies have shown that these mice display also co‐morbidities: cognitive and behavioral deficits, ataxia, SUDEP, dysregulated circadian rhythms, and sleep dysfunctions (Han et al., 2012; Ito et al., 2013; Mantegazza et al., 2021; Mantegazza & Broccoli, 2019).

Several other successive studies, including those performed with conditional mouse models that allow the expression of mutants in specific neuronal subtypes and with a *knock‐in* model of a GEFS+ mutation (*Scn1a* ^R1648H/+^), have confirmed that hypoexcitability of GABAergic neurons is the initial pathological mechanism in DS models and that the amount of LOF can determine the severity of the phenotype (Bryson & Petrou, 2023; Mantegazza et al., 2021; Mantegazza & Broccoli, 2019).

Notably, these *Scn1a*/Na_V_1.1 mouse models have also allowed to disclose genetic modifiers and cellular remodeling induced by the initial effect of the *Scn1a*/Na_V_1.1 mutation, which can modulate the phenotype‐inducing homeostatic or pro‐pathologic modifications, including homeostatic up‐regulation of Na^+^ channels in GABAergic neurons and seizure‐induced hyperexcitability of glutamatergic neurons (Hawkins et al., 2016; Mantegazza et al., 2021; Mantegazza & Broccoli, 2019; Mistry et al., 2014). For instance, the interaction between seizures and the genetic mutation can transform the basically asymptomatic phenotype of a *Scn1a* ^R1648H/+^ mouse model into a severe DS‐like one inducing remodeling (e.g., increased excitability of some excitatory glutamatergic neurons) (Salgueiro‐Pereira et al., 2019). Notably, detailed electrophysiological investigations show that excitability of glutamatergic neurons is not altered in different genetic backgrounds of this model when seizures are not experienced (Hedrich et al., 2014; Salgueiro‐Pereira et al., 2019). A further example of remodeling, in this case homeostatic, is the transient hypoexcitability reported in PV‐positive interneurons in layer II/III of the primary somatosensory cortex of a heterozygous knock‐out *Scn1a* model, which was observed only before P35, but not after that age, probably because of remodeling of the AIS (Favero et al., 2018). This study has been used to propose that hypoexcitability of GABAergic neurons and reduced GABAergic inhibition is not a pathological mechanism of DS at later stages of the disease. However, a recent study from the same group has shown that reduced GABAergic synaptic transmission of these neurons persists after P35 and depends on reduced axonal excitability (AP propagation), which is probably more sensitive to decreased sodium conductance than AP generation at AIS, as observed in computational modeling simulations (Kaneko et al., 2022). The only electrophysiological investigation of the activity of GABAergic neurons in vivo in *Scn1a* models was performed by juxtacellular recordings of cortical PV‐positive neurons from global *Scn1a* ^+/−^mice in the pre‐epileptic period, which did not show alterations, consistent with homeostatic responses (De Stasi et al., 2016). However, discharge frequency of the recorded neurons in this study was low, and alterations may be disclosed at a higher firing frequency.

Overall, these results show that the initial pathological mechanism in mouse models of epileptogenic *SCN1A* pathogenic variants is Na_V_1.1 LOF and hypoexcitability of at least some subtypes of GABAergic neurons. However, this initial dysfunction leads to and is accompanied by both homeostatic and pathologic remodeling, complex phenomena that depend on the type of cell, the age, the genetic background, and the interaction between Na_V_1.1 pathogenic variants and experienced seizures. More work is needed to shed light on this complex scenario.

#### 2.1.4 Therapeutic strategies

Although some drugs like stiripentol (Chiron et al., 2000), fenfluramine (Ceulemans et al., 2016), or cannabidiol (Devinsky et al., 2017) have been shown to be partially effective in some patients, DS still remains drug resistant for most of the patients. *Scn1a* gene‐targeted mice have been used for testing therapeutic approaches, both classical pharmacological treatments and novel methods, including gene therapy, in some cases obtaining significant amelioration of the phenotype, for a recent review see Mantegazza et al. (2021). Some novel approaches have shown particularly effective results and have led to clinical trials. For example, an antisense oligonucleotide (ASO)‐based targeted augmentation of nuclear gene output approach has been used to increase the expression of functional Na_V_1.1 channels in DS mice, observing a substantial decrease in spontaneous seizures and SUDEP when specific ASOs were injected in newborn mice (Han et al., 2020). These methods, including gene therapy, hold great promise for treatment of DS in children who may be identified by gene sequencing early in life before severe symptoms arise. Altogether, genetic therapeutic approaches have a high level of specificity for Na_V_1.1 channels and can reduce the effects of LOF, but the timing of treatment, method of delivery, and half‐life of therapeutic agents are still challenging problems.

Notably, *Scn1a* mutant zebrafish is a further animal model that, compared to mouse models, can be used for higher throughput screens (Baraban et al., 2013), although results obtained with this model have to be validated in mammalian models. Moreover, in vitro models of human neurons generated from iPS cells have been used to identify pathological mechanisms and test therapeutic approaches, but some technical problems related to variability and incomplete cellular maturation still limit their effectiveness, as recently reviewed (Mantegazza & Broccoli, 2019).

#### 2.1.5 GOF variants

Interestingly, *SCN1A*/Na_V_1.1 pathogenic variants involved in S/FHM cause Na_V_1.1 GOF by inducing numerous gating modifications and often by increasing the slowly inactivating “persistent” current fraction (Mantegazza et al., 2021; Mantegazza & Broccoli, 2019; Mantegazza & Cestele, 2018). This GOF was initially a puzzling finding in terms of pathogenic mechanism. Gene‐targeted mouse models of S/FHM *Scn1a*/Na_V_1.1 variants have been generated and they show facilitated/spontaneous generation of cortical spreading depolarization, a proposed pathological mechanism of migraine with aura, but no seizures (Auffenberg et al., 2021; Jansen et al., 2019). Acute models have shown that Na_V_1.1 GOF or optogenetic hyperactivation of GABAergic neurons leads to accumulation of extracellular K^+^ induced by hyperexcitability of GABAergic neurons, leading to generalized hyperactivity of cortical circuits and finally to concomitant depolarization block of both GABAergic and glutamatergic neurons (Chever et al., 2021; Desroches et al., 2019; Lemaire et al., 2021, 2023).

Notably, a very mild GOF has been observed for variants causing the NDEEMA‐EIDEE/MD‐EIDEE‐focal seizures spectrum (Berecki et al., 2019, 2023; Brunklaus et al., 2022; Matricardi et al., 2023). In fact, these recent studies, performed in transfected cell lines, have disclosed mild modifications of gating properties for these mutants, which do not show clear correlation with the phenotypes within the NDEEMA‐EIDEE/MD‐EIDEE‐focal seizures spectrum and are, in general, smaller than those observed for S/FHM *SCN1A*/Na_V_1.1 variants in the same experimental conditions (Brunklaus et al., 2022; Matricardi et al., 2023). A computational modeling study has proposed that homeostatic responses attempting to reduce hyperexcitability of PV‐positive GABAergic neurons induced by GOF NDEEMA‐EIDEE/MD‐EIDEE‐focal seizures variants may possibly induce instability in cortical network functions and be the pathological mechanisms of these variants (Berecki et al., 2023). However, this mechanism is still hypothetical and does not take into account that the larger GOF effects of S/FHM *SCN1A*/Na_V_1.1 variants should induce even larger homeostatic responses. Thus, more studies are needed to shed light on detailed pathological mechanisms.

Importantly, consistent with the GOF observed in functional studies, most of the patients in the NDEEMA‐EIDEE/MD‐EIDEE‐focal seizures spectrum respond to treatment with sodium channel blocker anti‐seizure medications, differently than patients that carry *SCN1A*/Na_V_1.1 LOF variants, whose phenotype is worsened by these drugs. Therefore, functional analysis of *SCN1A* pathogenic variants is important to tailor the appropriate therapeutic approach by discriminating GOF versus LOF ones.

### 2.2 *SCN2A/* Na_V_1.2

The *SCN2A* gene encodes the Na_V_1.2 sodium channel, which is widely expressed in the central nervous system, particularly in cortical and hippocampal glutamatergic neurons (Beckh et al., 1989; Mantegazza et al., 2021; Meisler et al., 2021). In rodents, Na_V_1.2 channels are the main Na_V_ of the AIS and the nodes of Ranvier in the first 10 days of postnatal life, then they are partially replaced by Na_V_1.6 and become mainly localized to the proximal AIS (Boiko et al., 2001; Kaplan et al., 2001; Liao et al., 2010). It has been proposed that proximal Na_V_1.2 channels promote backpropagation of APs to the soma (Hu et al., 2009). In adult rodents, Na_V_1.2 is also still present at relatively high density in thin neuronal processes, presumably distal unmyelinated portions of pre‐terminal axons.

#### 2.2.1 Phenotypic spectrum

**FIGURE 2:**
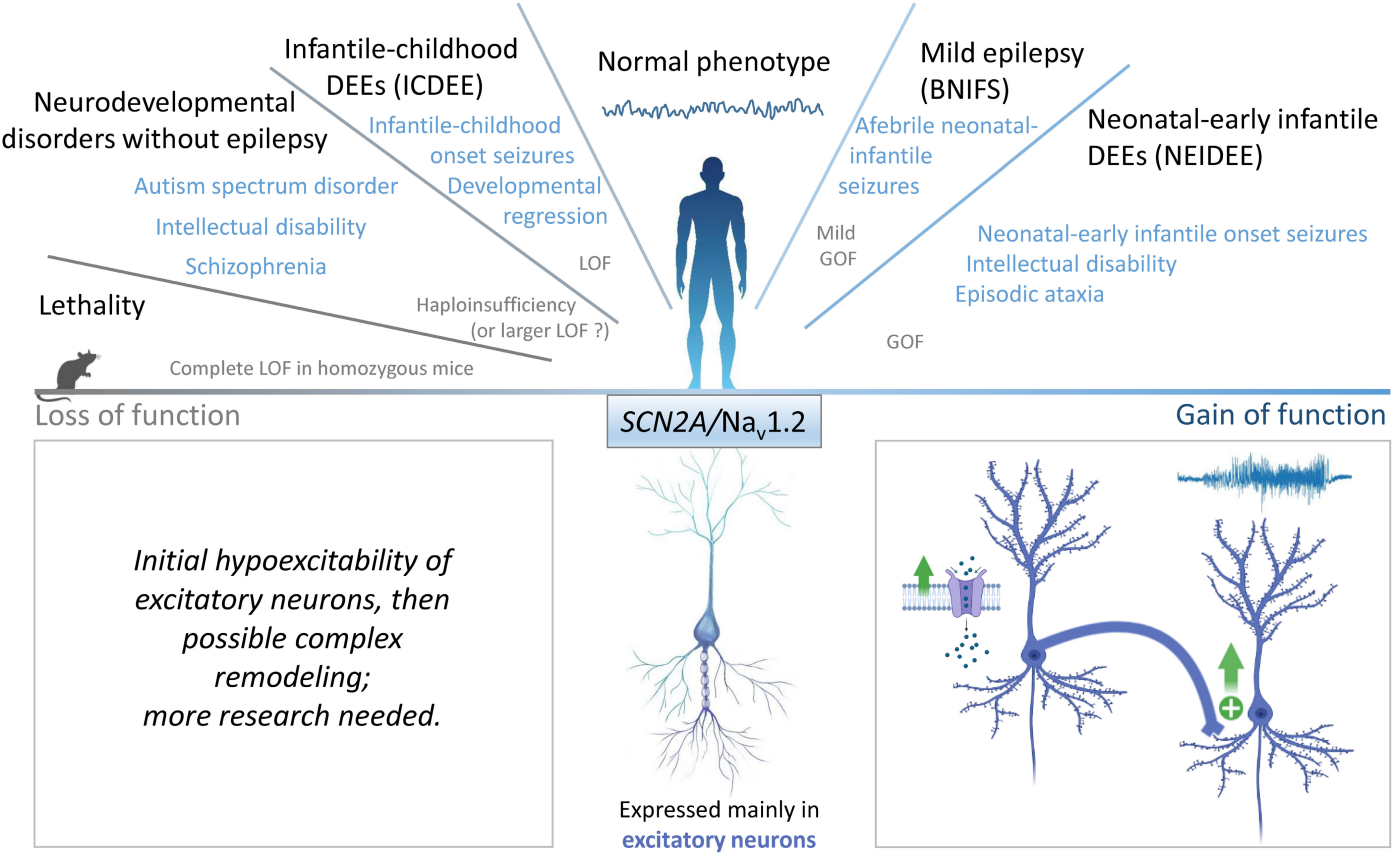
*SCN2A*/Na_v_1.2 variants: clinical phenotypes and functional effects. *Top panel*: phenotypic spectrum of *SCN2A* variants with correlation to functional effects. *Bottom panel*: (Middle) Na_v_1.2 is mainly expressed in excitatory neurons; (Left) pathological mechanisms of loss of Na_v_1.2 function are not yet clear; (Right) gain of Na_v_1.2 function in excitatory neurons can lead to network hyperexcitability.

Pathogenic variants of *SCN2A/*Na_V_1.2 can cause a wide phenotypic spectrum (Figure 2). The first epileptic syndrome clearly associated with these variants was benign familial neonatal/infantile seizures, characterized by a mild phenotype showing aFS with onset between 3 and 6 months of age and spontaneous remission within the first year of life, without subsequent neurological deficits (Heron et al., 2002). Subsequently, it has been shown that pathogenic variants of *SCN2A*/Na_V_1.2 cause a range of neurodevelopmental disorders, including DEE of varying severity. Pathogenic variants implicated in DEE generally arise de novo, and about 80% are missense. About 60% of *SCN2A/*Na_V_1.2 DEE have onset in the first 3 months of life, mostly in the neonatal period (Wolff et al., 2017, 2019). These neonatal–early infantile DEE patients show intellectual disability in all cases. About 50% of them have unclassifiable epileptic phenotypes with variable seizure types, whereas the others have phenotypes that fit the features of two epileptic syndromes: Ohtahara syndrome (neonatal‐onset spasms or tonic seizures and EEG with burst suppression pattern) or epilepsy with early infantile migrating focal seizures (multiple types of focal seizures that migrate from one hemisphere to the other). Early epileptic phenotypes include also epilepsy associated with episodic ataxia (Schwarz et al., 2019).

DEE with infantile and childhood onset are about 40% of the total (Wolff et al., 2017, 2019). The phenotypes of these patients can be correlated to the age of onset. Patients with onset between 3 months and 1 year of age, in general, show a West syndrome phenotype (infantile spasms as seizure type, hypsarrhythmic EEG, i.e., high amplitude waves on a background of multifocal irregular spikes, and developmental regression), with possible evolution into a Lennox–Gastaut phenotype (multiple seizure types, EEG with diffuse spike‐and‐wave and paroxysmal fast activity, and intellectual disability). Patients with onset after 1 year of age often show variable seizure phenotypes that cannot be classified as an established epileptic syndrome, including different types of seizures with developmental/cognitive delay and autistic traits that can appear before seizure onset. Other phenotypes with infantile and childhood onset include autism spectrum disorders (ASD) and/or intellectual disability, as well as other neuropsychiatric phenotypes such as schizophrenia (Sanders et al., 2018; Wolff et al., 2017, 2019). Notably, large‐scale human genetic studies have indicated that *SCN2A/*Na_V_1.2 variants are among those with the strongest association with ASD (Sanders et al., 2012; Satterstrom et al., 2020). Although seizures have been observed in some of these patients after the onset of neurodevelopmental dysfunctions, epilepsy is not a major feature of their phenotype.

#### 2.2.2 Functional effects

Functional analysis in transfected cell lines has shown interesting genotype–phenotype relationships (Mantegazza et al., 2021; Sanders et al., 2018). GOF consistent with neuronal hyperexcitability has been observed for *SCN2A/*Na_V_1.2 pathogenic variants identified in mild benign neonatal/infantile epilepsy (Scalmani et al., 2006) and neonatal/early infantile DEE (Wolff et al., 2017), whereas LOF consistent with hypoexcitability has been observed for pathogenic variants identified in infantile/childhood epileptic encephalopathy or neurodevelopmental disorders (e.g., autism and intellectual disability) without seizures (Wolff et al., 2017). Some of these features have been observed also in human neurons generated from iPS cells, although features related to the age of onset have not been thoroughly investigated with these models (Asadollahi et al., 2023; Que et al., 2021). However, it has been more difficult to identify genotype–phenotype relationships within categories of these GOF and LOF mutants, because functional effects of pathogenic variants causing different phenotypes often overlap. Overall, the results of these functional studies correlate well with the observation that patients with early onset of seizures respond better to sodium channel blocker anti‐seizure medications than those with later onset (Wolff et al., 2017). Interestingly, a study has found that phrixotoxin‐3, a sodium channel blocker that is relatively specific for Nav1.2, was particularly effective in reducing hyperexcitability in networks of human neurons expressing a GOF variant (Que et al., 2021).

#### 2.2.3 Animal models

Knock‐out *Scn2a* mice have been generated more than 20 years ago to investigate physiological functions of Na_V_1.2, observing that homozygous *Scn2a* ^−/−^ mice show perinatal mortality (Planells‐Cases et al., 2000). They have been re‐evaluated more recently and extensively investigated as a model of *SCN2A* diseases, in particular using heterozygous *Scn2a* ^+/−^ mice for mimicking LOF that leads to haploinsufficiency. Interestingly, investigations of neuronal features in brain slices of these models have shown impairment of intrinsic excitability in particular in the first days of life, consistent with the developmental pattern of Na_V_1.2 expression (Shin et al., 2019; Spratt et al., 2019). Notably, besides its established axonal role, it has been proposed that Na_V_1.2 can also have dendritic/synaptic functions in pyramidal neurons of the prefrontal cortex, where its haploinsufficiency impairs synaptic plasticity and strength, even when its expression is reduced only in sparse single neurons and late in postnatal development (Spratt et al., 2019). Notably, Na_V_1.2 haploinsufficiency did not induce the same synaptic remodeling in the hippocampus (Shin et al., 2019), consistent with effects that can be brain region specific. Overall, these studies have shown that *Scn2a* ^+/−^ mice, compared with *Scn1a* models, have a relatively mild phenotype, including short absence‐like seizures, spatial memory deficits but enhanced fear memory, some autistic and possibly schizophrenic features (Middleton et al., 2018; Ogiwara et al., 2018; Shin et al., 2019; Spratt et al., 2021). Notably, some features are age dependent; in particular, autistic traits are more prominent in young mice and tend to remit in adults (Léna & Mantegazza, 2019), consistent with the very mild autistic features reported in previous studies for adult mice. However, patients with LOF *SCN2A*/Na_V_1.2 pathogenic variants often have more prominent autistic traits, cognitive deficits, and epilepsy.

Interestingly, a more recent *Scn2a* mouse model with larger LOF than haploinsufficiency shows a more severe phenotype, in particular more robust autistic‐like features (Wang et al., 2021). Furthermore, two recent studies have shown that either complete deletion of *Scn2a* (*Scn2a* ^−/−^) in conditional floxed adult mice (Spratt et al., 2021) or reduction by gene trapping of *Scn2a* expression to about 20%–30% of that of the wild‐type condition (Zhang et al., 2021) can paradoxically induce hyperexcitability of glutamatergic neurons, although the proposed mechanisms of this counterintuitive results were different in the two studies: biophysical interaction with other ionic currents in one study (Spratt et al., 2021), down‐regulation of K^+^ currents in the other study (Zhang et al., 2021). Overall, reduction of *SCN2A*/Na_V_1.2 to more than haploinsufficiency could be needed to induce severe phenotypes, at least in mouse models, although detailed mechanisms are not clear yet.

Recently, a knock‐in mouse model carrying the recurrent *SCN2A*/Na_V_1.2 missense mutation R1882Q associated with early onset DEE has been generated (Li et al., 2021). Previous in vitro studies showed that R1882Q causes GOF, with increased peak and persistent sodium currents that induce an increase of the AP firing frequency in dynamic clamp experiments (Berecki et al., 2018). *Scn2a* ^*R1882Q/+*^ mice develop spontaneous seizures at P1 and suffer premature death, thus recapitulating the severe phenotypes observed in patients. This mouse model was used to develop a targeted gene therapy based on ASOs to down‐regulate *Scn2a* mRNA expression. *Scn2a* ASO‐mediated knockdown by intracerebroventricular injections effectively reduced premature death and spontaneous seizures in *Scn2a* ^*R1882Q/+*^ mice. Also, the *Scn2a* ^*R1882Q/+*^ mice treated with moderate dose of anti‐*Scn2a* ASO showed behavioral features that were similar to those of WT mice.

Overall, available studies have shown that GOF *SCN2A*/Na_V_1.2 variants induce pathological mechanisms that are consistent with increased excitability of excitatory neurons, whereas LOF variants show more intricate mechanisms, which initially can be consistent with reduced excitability of excitatory neurons, but may then lead to complex remodeling of the functions of neurons and neuronal networks (Figure 2).

### 2.3 *SCN3A/* Na_V_1.3

The *SCN3A* gene encodes the Na_V_1.3 Na^+^ channel, whose functions have not been completely determined yet. Na_V_1.3 is widely expressed in the brain at high levels during embryonic development, but postnatal expression is very low in both rodents and humans (Beckh et al., 1989; Mantegazza et al., 2021; Meisler et al., 2021; Smith et al., 2018). Its involvement in human disease was not clearly established.

**FIGURE 3:**
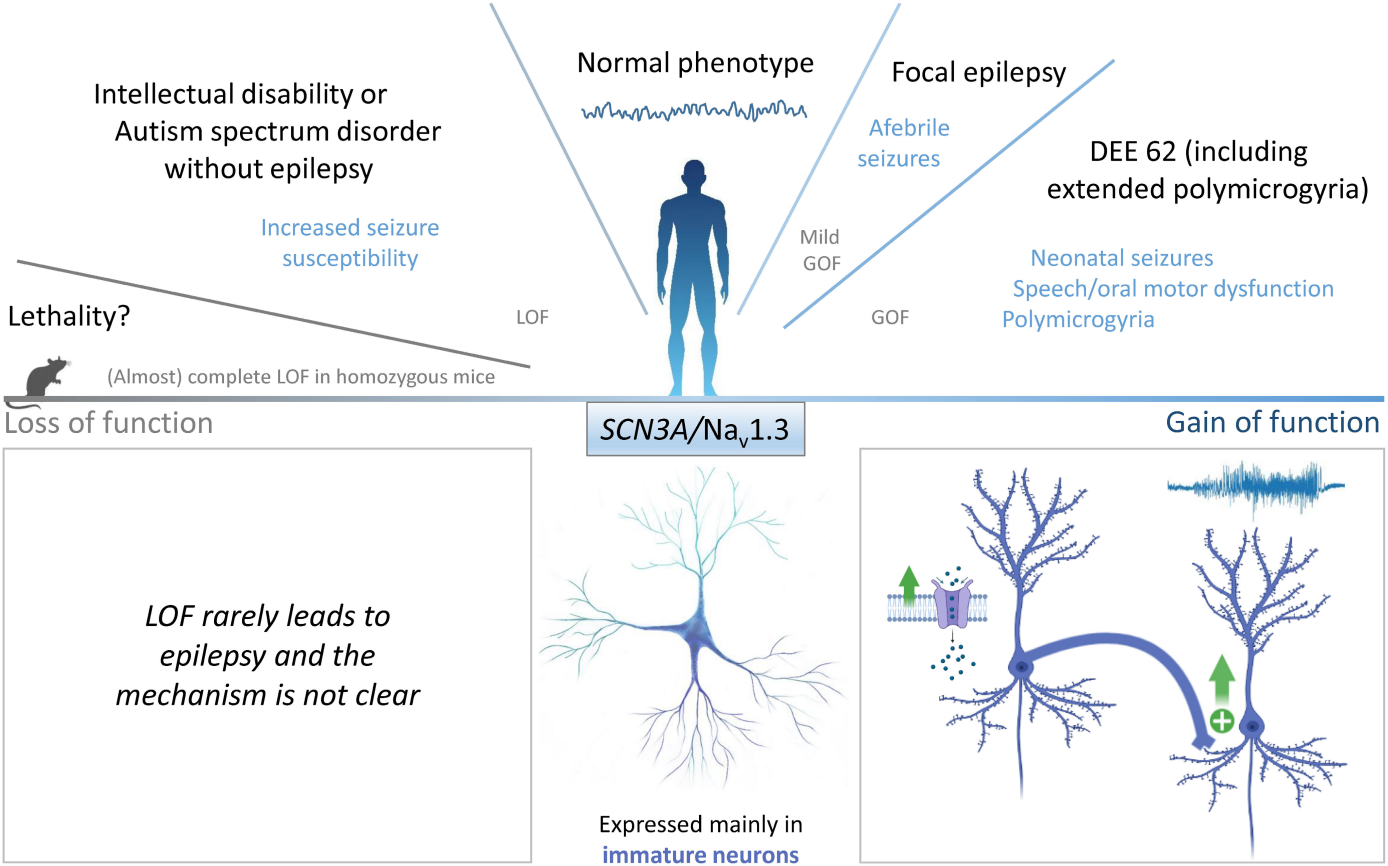
*SCN3A*/Na_v_1.3 variants: clinical phenotypes and functional effects. *Top panel*: Phenotype spectrum of *SCN3A* variants with correlation to functional effects. *Bottom panel*: (Middle) Na_v_1.3 is mainly expressed in immature neurons; (Left) loss of Na_v_1.3 function rarely leads to epilepsy and the pathological mechanism is not yet clear; (Right) gain of Na_v_1.3 function in immature neurons may lead to hyperexcitability.

Recently, de novo heterozygous pathogenic variants of *SCN3A/*Na_V_1.3 have been identified in an early infantile DEE (Figure 3) characterized by multifocal seizures, severe intellectual disability and, for some cases, polymicrogyria (multiple small gyri creating excessive folding of the cerebral cortex) (Zaman et al., 2018), as well as in families showing speech and oral motor dysfunctions associated with polymicrogyria of the perisylvian cerebral cortex but that did not typically show epilepsy (Smith et al., 2018). Disrupted cerebral cortical folding and neuronal migration were recapitulated in ferrets expressing the mutant Na_V_1.3, complementing previous work on mice and rats, which do not have cortical gyri (Smith et al., 2018). These studies in ferrets disclose a possible role of *SCN3A* in progenitor cells and migrating neurons involved in prenatal development of human cortical language areas.

A further more recent study (Zaman et al., 2020), performed with a larger cohort of patients, showed that *SCN3A*‐related clinical phenotypes show a wide spectrum, including mild epilepsy with intellectual dysfunction, early infantile DEE often associated with polymicrogyria, as well as a phenotype of speech and oral motor dysfunctions associated with polymicrogyria without epilepsy; ictal and non‐ictal autonomic dysfunction or microcephaly can be additional clinical features. Relatively mild neonatal–childhood focal epilepsy (Vanoye et al., 2014) would be the mildest phenotype in the spectrum. Onset is scattered between the neonatal and the early childhood period for all the phenotypes.

Functional studies have been performed in transfected cell lines (Smith et al., 2018; Zaman et al., 2018, 2020). They have shown that most of the *SCN3A* pathogenic variants identified in patients with severe phenotypes exhibit prominent GOF consistent with hyperexcitability, inducing in particular large increases of persistent sodium current, whereas variants identified in patients with milder phenotypes exhibit less pronounced GOF consistent with hypoexcitability. A puzzling finding is that LOF caused by reduced current density has been reported for some *SCN3A* variants. Heterozygous adult *Scn3a* ^+/−^ gene‐trap mice were investigated as a model of *SCN3A* LOF pathogenic variants, but they do not show DEE features. In fact, they have increased susceptibility to induced seizures and deficits in locomotor activity, but no spontaneous seizures or abnormalities in other behavioral features (Lamar et al., 2017). Homozygous *Scn3a* ^−/−^ gene‐trap mice, which maintain some Na_V_1.3 expression, have been characterized in less detail; increased mortality has been reported (Lamar et al., 2017).

Most *SCN3A*‐positive patients are drug resistant, including patients who carry GOF pathogenic variants and that are resistant to antiepileptic treatment with sodium channel blockers. Perhaps earlier initiation of therapy and higher doses may be more effective.

### 2.4 *SCN8A/* Na_V_1.6

The *SCN8A* gene encodes Na_V_1.6, the main sodium channel in the AIS and nodes of Ranvier of myelinated axons of mature excitatory neurons (Mantegazza et al., 2021; Meisler et al., 2021).

#### 2.4.1 Phenotypic spectrum

**FIGURE 4:**
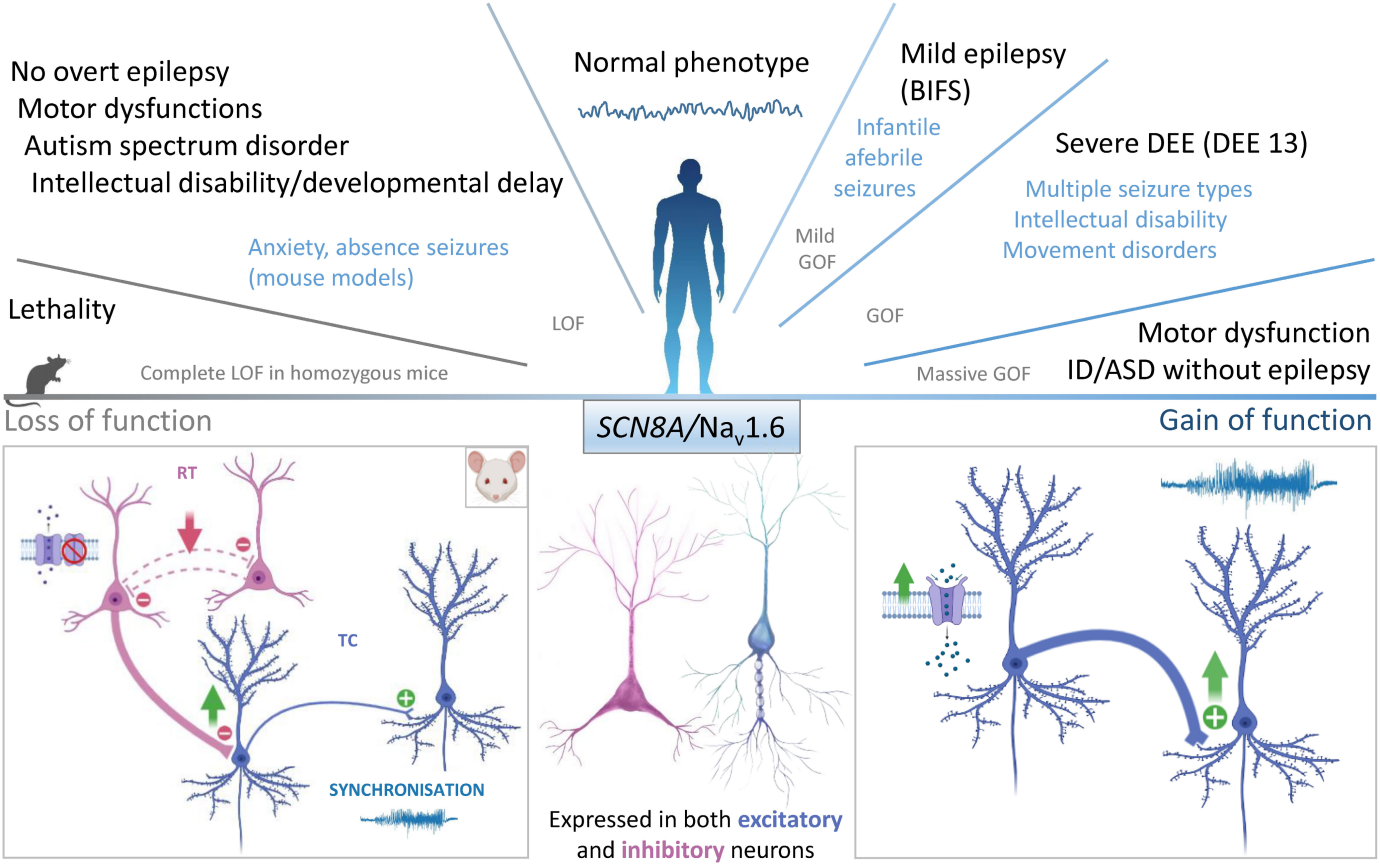
*SCN8A*/Na_v_1.6 variants: clinical phenotypes and functional effects. *Top panel*: Phenotype spectrum of *SCN8A* mutations with correlation to functional effects. *Bottom panel*: (Middle) Na_v_1.6 is expressed in both excitatory and inhibitory neurons; (Left) Example of a proposed loss of Na_v_1.6 function mechanism in the inhibitory interneurons of the reticular thalamus, causing reciprocal interneurons' disinhibition that produces prolonged synchronized inhibitory output affecting the timing of thalamocortical oscillations and leading to network spikes and wave discharges (absence‐like phenotype) in *Scn8a* haploinsufficient mice; however, no epileptic activity has been observed in patients yet; (Right) gain of Na_v_1.6 function in excitatory neurons can lead to network hyperexcitability.

De novo heterozygous pathogenic variants of *SCN8A/*Na_V_1.6 have been identified in a range of phenotypes (Figure 4). Most patients show DEE13, which is characterized by early onset epilepsy with multiple seizure types, infrequent FS, EEG abnormalities mainly in the temporo‐occipital regions, severe intellectual disability, and movement disorders (Gardella et al., 2018). Other patients show milder phenotypes, including benign familial infantile seizures with paroxysmal dyskinesia (Gardella et al., 2016), and epilepsies with intermediate phenotypes (Johannesen et al., 2019). Furthermore, some patients show intellectual disability, autism, or movement disorders without epilepsy (Liu et al., 2019; Wagnon et al., 2017). Notably, >20% of patients have recurrent pathogenic variants.

#### 2.4.2 Functional effects

Pathogenic variants that cause DEE13 or milder epilepsy induce GOF and include negative shifts of voltage dependence of activation, positive shifts of voltage dependence of inactivation, slowed channel inactivation, or increased persistent or resurgent current. These functional changes are all consistent with neuronal hyperexcitability, which has been observed in the studies that evaluated the effect on AP discharges of transfected cultured neurons (Liu et al., 2019; Veeramah et al., 2012). Other pathogenic variants causing intellectual disability, autism, or movement disorders without epilepsy induce LOF, which can lead to haploinsufficiency (Liu et al., 2019; Wagnon et al., 2017). Interestingly, massive GOF can induce reduced generation of APs, as disclosed by expression in transfected cultured neurons, mimicking LOF pathogenic variants and inducing a phenotype that is similar to that of LOF pathogenic variants (Liu et al., 2019).

#### 2.4.3 Animal models

Mouse models of *SCN8A/*Na_V_1.6 DEE pathogenic variants are available: a standard global knock‐in of the N1768D variant (Wagnon et al., 2015) and a conditional floxed knock‐in of the R1872W variant (Bunton‐Stasyshyn et al., 2019). Both mice, in heterozygosis, recapitulate some features of DEE13 presenting with spontaneous seizures, SUDEP, and mild impairment of motor coordination. Electrophysiological recordings showed increased Na^+^ currents and hyperexcitability of excitatory neurons. Conditional floxed mice confirmed that properties of excitatory neurons are mainly modified, because selective expression of the variant in excitatory neurons induced a phenotype that was similar to that of global expression, whereas selective expression of the variant in inhibitory GABAergic neurons did not induce an overt phenotype (Bunton‐Stasyshyn et al., 2019). Overall, knock‐in mice have confirmed that GOF pathogenic variants of *SCN8A* are sufficient to induce hyperexcitability of some subtypes of excitatory neurons, generating severe seizures and mortality, similar to the clinical features of *SCN8A/*Na_V_1.6 DEE. In contrast, spontaneous mouse models carrying LOF Na_V_1.6 pathogenic variants (Meisler et al., 2004) show a phenotype that is similar to that of patients with intellectual disability and/or movement disorders without prominent epilepsy. Non‐convulsive absence seizures have been observed in heterozygous *SCN8A/*Na_V_1.6 knock‐out mice, and floxed models with selective knock‐out in selective neurons have shown that they are generated by reduced desynchronizing recurrent synaptic inhibition in neurons of the thalamic reticular *nucleus* leading to prolonged synchronized inhibitory output affecting the timing of thalamocortical oscillations and to spikes and wave discharges (absence‐like phenotype) (Makinson et al., 2017).

#### 2.4.4 Therapeutic strategies

Some patients with *SCN8A* pathogenic variants respond to high doses of sodium channel blocker drugs (Boerma et al., 2016), but available blockers for clinical use are not isoform‐specific and chronic therapy at high doses can induce adverse effects. In a recent study, conditional knock‐in mice carrying a GOF variant (*Scn8a* ^COND_R1872W/+^) were treated with ASOs that reduce *Scn8a/*Na_V_1.6 expression by 25%–50%, showing a delay of spontaneous seizure onset and lethality (Lenk et al., 2020). Interestingly, the same treatment was effective also for *Scn1a* ^+/−^ mice, a Dravet syndrome model. These genetic approaches are highly specific for Na_V_1.6, but problems of drug delivery and drug half‐life have to be solved for translational use. Interestingly, a further study reported the characterization of a novel selective Na_V_1.6 inhibitor, the small molecule NBI‐921352, showing that it inhibits firing of pyramidal excitatory neurons but spares fast‐spiking interneurons (Johnson et al., 2022). Acute oral administration of NBI‐921352 (which is rapidly cleared in mice complicating chronic treatments and evaluation of the effect on spontaneous seizures) inhibited electrically induced seizures in both *Scn8a* ^N1768D/+^ and wild‐type mice (Johnson et al., 2022). Clinical trials are evaluating NBI‐921352 in SCN8A‐DEE patients and adult patients with focal onset seizures.

## 3 ACCESSORY β SUBUNITS

β subunits were originally denominated accessory because they do not directly form the channel, but can modulate the properties of α subunits. There are four genes (*SCN1B* to *SCN4B)* that encode for four β subunit isoforms (β1 to β4), which are widely expressed in different organs, including the central nervous system. We now know that they are multifunctional molecules implicated, besides direct modulation of α subunits, in diverse roles in multiple tissues, including cell adhesion, cell migration, neuronal pathfinding, fasciculation, and neurite outgrowth (Bouza & Isom, 2018). Thus, pathogenic variants of β subunits can alter numerous functions, including modulations of all the α subunits, and are involved in different diseases (Bouza & Isom, 2018). *SCN1B/*β1 is the only β subunit whose genetic variants have been implicated in epilepsy.

The heterozygous missense mutation C121W, identified in a patient with mild Genetic Epilepsy with Febrile Seizures plus (GEFS+), was among the first epileptogenic pathogenic variants identified (Wallace et al., 1998). Homozygous *SCN1B*/β1 pathogenic variants have been later identified in DEE patients that were initially included in the Dravet syndrome spectrum (Patino et al., 2009). However, it is now clear that their clinical features are distinct from those of Dravet syndrome, showing earlier onset and more severe phenotype, including early infantile‐onset seizures, psychomotor stagnation or regression, microcephaly, axial hypotonia, appendicular spasticity, and nonspecific brain atrophy (Aeby et al., 2019; Scala et al., 2021). In some cases, developmental delay is observed before seizure onset, consistent with more prominent developmental features in the *SCN1B/*β1 DEE phenotype (Aeby et al., 2019; Scala et al., 2021). Moreover, *SCN1B/*β1 pathogenic variants have not been identified in typical Dravet syndrome patients (Kim et al., 2013).

Functional analysis of *SCN1B/*β1 DEE pathogenic variants in transfected cell lines has identified either LOF (loss of functional modulation of co‐expressed α subunits) or induction of complex gating modifications in different co‐expressed α subunits (Aeby et al., 2019; Patino et al., 2009; Scala et al., 2021). Notably, little is known about the effect of these pathogenic variants on the other functions of β subunits that are not directly linked to modulation of α subunits.

Homozygous knock‐out null *Scn1b* ^−/−^ mice, which show spontaneous seizures and high mortality rate, have been investigated as model of *SCN1B/*β1 DEE (Hull et al., 2020). Recordings of the firing of cortical neurons in brain slices obtained from these mice have shown dysfunctions in the excitability of both pyramidal excitatory neurons and GABAergic fast‐spiking interneuron. In fact, GABAergic interneurons were hypoexcitable, whereas dysfunctions of pyramidal neurons were more complex, with subsets of pyramidal neurons exhibiting hyperexcitability at low current injections and hypoexcitability at high stimulation intensities.

## 4 OTHER INTERACTING PROTEINS

The Na_V_ core complex, formed by α and β subunits, can interact with numerous other proteins, which are not exclusive partners, forming heterogeneous multimolecular complexes that can be specific to different cell types or different cell sub‐compartments (Curran & Mohler, 2015; Dib‐Hajj & Waxman, 2010; Terragni et al., 2018). As already highlighted, these interactions can be important for trafficking and localization of the core channel complex, as well as for modulation of its functional properties. They can also be implicated in rescuing folding/trafficking defective pathogenic mutants (Bechi et al., 2015; Rusconi et al., 2007, 2009; Terragni et al., 2018). Genetic variants of some interacting proteins that have an effect on Na_V_ have been identified in epileptic phenotypes. We will review here some relevant examples.

### 4.1 FGF12/FHF1

The fibroblast growth factor homologous factor (FGF/FHF) family, composed of four members, has sequence and structure that is related to fibroblast growth factors (FGFs), but function that is unrelated (Goldfarb, 2005). In fact, classical FGFs bind to the extracellular domain of cell surface receptor tyrosine kinases, whereas *FGF/FHF* are small intracellular proteins. They can bind to the C‐terminal cytoplasmic domain of Na_V_ α subunits and modulate their gating, increasing sodium currents and excitability (Goldfarb et al., 2007). Expression of the four FGF/FHF genes begins in neurons during embryogenesis and persists through adulthood, with individual neurons expressing distinct repertoires of FHF transcripts. Immunocytochemistry data show strong localization of FGF/FHF to nodes of Ranvier along myelinated axons.

Pathogenic variants of *FGF12/FHF1* have been identified in DEE patients, initially as de novo heterozygous missense variants (Siekierska et al., 2016) and more recently also as chromosomal microduplications involving the entire gene (Oda et al., 2019). The phenotype of *FGF12/FHF1* DEE comprises intractable seizures with onset in the first days or weeks of life, intellectual disability, and behavioral disturbances with mild cerebral and/or cerebellar atrophy observed with brain magnetic resonance imaging (Trivisano et al., 2020).

Functional studies performed in transfected cell lines showed a GOF effect of missense pathogenic variants, with mutant *FGF12/FHF1* that induced a larger positive shift of the inactivation curve of co‐expressed *SCN8A*/Na_V_1.6 compared to WT, leading to increased current that is consistent with increased neuronal excitability (Siekierska et al., 2016). Transgenic over‐expression of a mutant *FGF12/FHF1* in zebrafish larvae enhanced epileptiform discharges, demonstrating the effect of the mutation in vivo (Siekierska et al., 2016). Notably, *FGF12/FHF1* duplication may induce a GOF effect that is analogous to that of missense pathogenic variants.

Knock‐out null *Fgf12/Fhf1* ^*−/−*^ mice show severe ataxia and hypoexcitability of cerebellar granule neurons. Recently, knock‐in mice of the *FGF12/FHF1* DEE missense mutation R52H have been generated and heterozygous *Fgf12/Fhf1* ^*R52H/*+^ mice show spontaneous seizures and mortality between 12 and 26 days of age (Veliskova et al., 2021).

### 4.2 PRRT2

Proline‐rich transmembrane protein‐2 (PRRT2) is neuron specific and is targeted to synaptic and axonal domains, where it can modulate intrinsic excitability and synaptic transmission (Valtorta et al., 2016). Among other targets, PRRT2 negatively modulates Na_V_1.2/1.6 channels by decreasing their targeting to the plasma membrane and enhancing inactivation, but it does not modulate Na_V_1.1 channels (Fruscione et al., 2018). Thus, it inhibits intrinsic neuronal excitability, particularly in excitatory neurons. PRRT2 has been identified as the causative gene for a spectrum of paroxysmal neurological disorders including mainly Benign Familial Infantile Epilepsy, Paroxysmal Kinesigenic Dyskinesia (PKD), and Paroxysmal Kinesigenic Dyskinesia with Infantile Convulsions, with fewer patients presenting with episodic ataxia or hemiplegic migraine (Doring et al., 2020; Riant et al., 2022). Relatively rare patients show a severe DEE phenotype and carry compound heterozygous or homozygous variants (Guerrini et al., 2023). Most variants cause truncations of PRRT2 with consequent LOF (Doring et al., 2020; Guerrini et al., 2023). The increased function of Na_V_ channels seems to be a central pathological mechanism of disorders linked to PRRT2 LOF because sodium channel blockers can control symptoms in PRRT2 patients, who are not responsive to other anti‐seizure medications that have different targets (Chou et al., 2014; Doring et al., 2022; Suzuki‐Muromoto et al., 2020). Homozygous knock‐out *PRRT2* mice reproduce human phenotypes showing increased sensitivity to convulsants, paroxysmal movements at the onset of locomotion, and abnormal motor behaviors, reproducing some human phenotypes (Michetti et al., 2017). Interestingly, not only PRRT2 LOF is involved in pathologies: it has been recently reported that a missense variant involved in PKD can induce PRRT2 GOF (i.e., enhanced inhibition of Na_V_1.2 channels) (Sterlini et al., 2023).

### 4.3 ANKYRIN‐G

Ankyrin proteins are molecular scaffolds and Ankyrin‐G, encoded by the *ANK3* gene, plays an essential role in the localization of voltage‐gated ion channels, including Na_V_s, to specialized neuronal plasma membrane subdomains, in particular the AIS and nodes of Ranvier (Nelson & Jenkins, 2017). ANK3 variants have been implicated in bipolar disorder (BD), schizophrenia, and ASD (Yoon et al., 2022). In particular, a homozygous truncating mutation has been reported in a family with developmental delay, intellectual disability, behavioral abnormalities, and, in one member, epilepsy (Iqbal et al., 2013). Interestingly, *ANK3* exon 1b isoform is expressed mainly in GABAergic interneurons and knock‐out mice deficient for exon 1b show reduced Na_V_ channels at the AIS of parvalbumin‐positive interneurons (which have increased firing thresholds and diminished AP dynamic range), behavior dysfunctions modeling BD, epilepsy, and sudden death (Lopez et al., 2017).

## 5 CONCLUSIONS

The past two decades have witnessed a wide range of studies that have investigated genetic variants of Na_V_ channels, which are involved in a broad spectrum of diseases, including several types of epilepsy. As we have outlined here, this effort has shed some light on the clinical phenotypes and the pathological mechanisms involved, allowing us to better understand pathophysiological issues, ameliorate diagnostics, orientate genetic counseling, and select/develop therapies within a precision medicine framework. These studies have also allowed us to gain insights into the physiological roles of different Na_V_ channels and of the cells that express them. Overall, our review shows the progress that has been made, but also the need for further studies of issues that have yet to be clarified. Moreover, it highlights several significant general themes.

### 5.1 Both GOF and LOF can cause disease

It was initially often thought that the preponderant dysfunction of Na_V_ channels in epilepsy was an increase in their function because this appeared consistent with neuronal hyperexcitability and this dysfunction is opposite to the effect of sodium channel blockers, which are anti‐seizure medications. However, LOF was instead the predicted effect of the first Na_V_ genetic variants (of Na_V_1.1) clearly linked to a well‐defined epileptic phenotype (Dravet syndrome) (Claes et al., 2001). It is now clear that both GOF and LOF of all the Na_V_ channels can cause disease. Although detailed pathological mechanisms at the level of cellular and network functions are not always clear yet, the identification of the functional effect at the channel level is very important to orientate research efforts and to select therapies (e.g., use or non‐use of sodium channel blockers). Overall, this is consistent with the existence of a physiological window of function outside of which, in any direction, a pathological state is induced.

### 5.2 Small effects are important

This physiological window can be narrow. In fact, small functional effects, such as modifications of gating properties by only a few percent, are sufficient in some cases to be pathogenic. For some phenotypes, there is a loose linear relationship between extent of functional effect and phenotype severity. However, this is not a general rule, as observed for Na_V_1.1 GOF variants that can cause extremely severe phenotypes but small functional effects (Berecki et al., 2019; Brunklaus et al., 2022; Matricardi et al., 2023).

### 5.3 Detailed functional analysis is critical and should be included in the clinical workup

Predictive tools can help, in some cases, in inferring pathogenicity and functional effect of genetic variants. However, their reliability is often limited, and functional electrophysiological investigations are still the gold standard and are the only method for disclosing detailed functional effects of some variants (Mantegazza & Cestele, 2022). This is the only reliable method available for gaining insights into the complex biophysical properties of ion channels, thus providing a basis for targeting specific dysfunctions with drugs in order to rescue the function of mutants. Functional tests have been extensively used in basic research, but it would be important to integrate them into the standard clinical workup. For this goal, it is essential to optimize the pipeline minimizing the complexity of functional tests and the time needed to execute them, as well as standardize experimental conditions and data interpretation in order to obtain robust functional evaluations. In particular, it will be important to use the correct cDNA clones, to express mutants in relevant cell types, and to perform experiments for investigating overall effects of variants, including the overall effect on neuronal excitability.

### 5.4 Effects in neurons can be complex

One might think that it is relatively straightforward to translate GOF and LOF at the channel level to increased or decreased (up and down) excitability at the neuronal level. However, effects on neurons can be complex and surprising. For example, some variants causing LOF in cell lines can be rescued by the neuronal cell background and may be transformed into GOF (Cestele et al., 2013; Dhifallah et al., 2018). Moreover, variants causing large GOF may be equivalent at the cellular level to those that cause large loss of function, because they can induce depolarization block of excitability (Liu et al., 2019). Notably, the final overall effect may depend on the neuronal subtypes, which may induce differential rescue or be more or less impacted by their properties, as shown in modeling studies (Koch et al., 2023). Thus, the expression of mutants in relevant subtypes of neurons is very important for functional studies, at least for some variants.

### 5.5 Animal models are essential

Although it can provide important information on the initial direct functional effect of a mutation, analysis of functional effects of Na_V_ variants in vitro is insufficient. In fact, an additional level of complexity has to be taken into account, because although the simple GOF‐LOF and hyperexcitability–hypoexcitability dychotomies are useful, they may not always be sufficient to understand detailed pathological mechanisms and identify effective therapies, which may depend on complex network dysfunctions that are not easily identified in reduced systems. Additionally, although the genetic variant is the initial pathogenic cause, the alteration of the biological system induced by its initial functional effect may induce complex homeostatic and pro‐pathological responses, whose identification requires model organisms. Moreover, animal models are essential for testing therapeutic approaches on complex in vivo features (seizures, behavioral dysfunctions, etc.). However, careful comparisons and critical evaluations of human and animal phenotypes are necessary to integrate phenotypic features and age‐related specificities of diseases in different species.

### 5.6 The genetic background can modulate the effects of variants

The initial direct functional effect of a genetic variant can be modulated both qualitatively and quantitatively by individual genetic background, as it has been demonstrated in mouse models, which can be implicated in the phenotypic variability that is often observed in patients. The use of human neurons, in particular neurons obtained with induced pluripotent stem cell (iPSC) technologies, has the potential to complement phenotypic studies. However, technical problems related to variability and incomplete cellular maturation have still to be solved and limit their effectiveness (Mantegazza & Broccoli, 2019). Moreover, at this stage, iPSC technologies are too time consuming and expensive to be used in standard clinical pipelines.

## AUTHOR CONTRIBUTIONS

Evgeniia Rusina, Martina Simonti, and Massimo Mantegazza prepared figures; Evgeniia Rusina, Martina Simonti, Sandrine Cestèle, and Massimo Mantegazza drafted the manuscript; Evgeniia Rusina, Martina Simonti, Fabrice Duprat, Sandrine Cestèle, and Massimo Mantegazza edited and revised the manuscript; all the authors approved the final version of the manuscript. We thank all the members of our laboratory who have contributed to obtain the results presented in this review.

## CONFLICT OF INTEREST STATEMENT

We declare no conflicts of interest.

### PEER REVIEW

The peer review history for this article is available at https://www.webofscience.com/api/gateway/wos/peer‐review/10.1111/jnc.15947.

## Data Availability

The data presented are available from the authors of the original articles.
